# The phylogeography and ecology of *Oligobrachia* frenulate species suggest a generalist chemosynthesis-based fauna in the arctic

**DOI:** 10.1016/j.heliyon.2023.e14232

**Published:** 2023-03-02

**Authors:** Arunima Sen, Liselotte W. Andersen, Kasper U. Kjeldsen, Loïc N. Michel, Wei Li Hong, Marvin Choquet, Tine L. Rasmussen

**Affiliations:** a Department of Arctic Biology, The University Centre in Svalbard (UNIS), Longyearbyen, Norway; b Faculty of Biosciences and Aquaculture, Nord University, Bodø, Norway; c Department of Biology, Section for Microbiology, Aarhus University, Aarhus, Denmark; d Department of Ecoscience, Aarhus University, Aarhus, Denmark; e Univ Brest, CNRS, Ifremer, UMR6197 BEEP (Biologie et Ecologie des Ecosystèmes Marins Profonds), Plouzané, France; f Department of Geological Sciences, Stockholm University, Stockholm, Sweden; g Department of Medical Biochemistry and Microbiology, Uppsala University, Uppsala, Sweden; h Centre for Arctic Gas Hydrate, Environment and Climate (CAGE), Department of Geosciences, The Arctic University of Norway, Tromsø, Norway

**Keywords:** Siboglinids, Seeps, Fjords, Ancient DNA, Sulfide oxidation, Methane

## Abstract

We used ancient DNA (aDNA) extraction methods to sequence museum voucher samples of *Oligobrachia webbi,* a frenulate siboglinid polychaete described from a northern Norwegian fjord over fifty years ago. Our sequencing results indicate a genetic match with the cryptic seep species, *Oligobrachia haakonmosbiensis* (99% pairwise identity for 574 bp mtCOI fragments)*.* Due to its similarity with *O. webbi*, the identity of *O. haakonmosbiensis* has been a matter of debate since its description, which we have now resolved. Furthermore, our results demonstrate that chemosynthesis-based siboglinids, that constitute the bulk of the biomass at Arctic seeps are not seep specialists. Our data on sediment geochemistry and carbon and nitrogen content reveal reduced conditions in fjords/sounds, similar to those at seep systems. Accumulation and decomposition of both terrestrial and marine organic matter results in the buildup of methane and sulfide that apparently can sustain chemosymbiotic fauna. The occurrence of fjords and by extension, highly reducing habitats, could have led to Arctic chemosymbiotic species being relatively generalist with their habitat, as opposed to being seep or vent specialists. Our stable isotope analyses indicate the incorporation of photosynthetically derived carbon in some individuals, which aligns with experiments conducted on frenulates before the discovery of chemosynthesis that demonstrated their ability to take up organic molecules from the surrounding sediment. Since reduced gases in non-seep environments are ultimately sourced from photosynthetic processes, we suggest that the extreme seasonality of the Arctic has resulted in Arctic chemosymbiotic animals seasonally changing their degree of reliance on chemosynthetic partners. Overall, the role of chemosynthesis in Arctic benthos and marine ecosystems and links to photosynthesis may be complex, and more extensive than currently known.

## 1 Introduction

Chemosynthesis-based ecosystems among them, hydrocarbon seeps and hydrothermal vents, are unique among deep-sea benthic ecosystems because food production and carbon fixation are sourced locally by energy derived through the oxidation of reduced compounds released from the geosphere [[1], [2], [3], [4]]. Though a plethora of reduced compounds can fuel chemosynthesis, two common energy sources are sulfide and methane. The capacity for chemolithoautotrophy (chemosynthesis) is limited to microbes (both archaea and bacteria), however chemosynthetic bacteria often form symbiotic associations with animals. The degree of nutritional dependence between an animal host and its chemosynthetic symbionts, and the closeness of partnerships, ranges from feeding on bacteria colonizing the surfaces of external appendages, to highly specialized, obligate intracellular relationships where host species have lost feeding and digestive organs entirely [1,[5], [6], [7], [8]]. Sulfide-based symbioses require further adaptations on the part of host species because sulfide is extremely toxic due it its ability to block oxidative respiration [[9], [10], [11], [12]]. This means that fauna that harbor sulfide-oxidizing chemosynthetic partners face the irony of requiring costly adaptations to mitigate the lethality of the very compounds that serve as their lifeline. As a result, highly reducing habitats such as vents and seeps tend to host specialist fauna and in particular, obligate chemosynthesis-based symbioses are often restricted to such systems [4,13,14].

In high latitude regions where past ice ages have carved fjords into coastlines with moraine deposits and/or sills limiting water exchange with the open ocean, organic matter accumulation and decomposition can lead to an appreciable buildup of sediment methane and sulfide [[15], [16], [17], [18], [19], [20]]. As a result, chemosynthesis-based animals, such as thyasirid bivalves and siboglinid frenulates have been commonly recorded in such fjords [[21], [22], [23]]. In fact, most of the early research on siboglinid frenulates was conducted among species from Norwegian fjords, long before chemosynthesis as a metabolic pathway was even discovered [21,[24], [25], [26], [27], [28]]. Therefore, chemosymbiotrophic fauna are quite widespread in Arctic and subarctic regions. This means that animal-host associated chemosynthetic production is a common part of Arctic benthic ecosystems, though this, and its contribution towards carbon and energy transfer, has been largely overlooked.

If chemosymbiotrophic animals are widely distributed in the Arctic, the question that then arises is whether seep or vent specialist chemosymbiotrophic fauna occur in the Arctic at all. The only confirmed chemosymbiotic animal that is present at Arctic vents is *Sclerolinum contortum* [[29], [30], [31]], a moniliferan siboglinid known to not be a seep or vent specialist [32,33]. This species is also found at the Haakon Mosby Mud Volcano (HMMV), the first Arctic seep to be studied. However, another dominant faunal community member at HMMV is a siboglinid frenulate species of *Oligobrachia* which at the time was described as a novel species new to science and named after the site (*Oligobrachia haakonmosbiensis*) [34]. Since frenulates can be seep specific (e.g., *Siboglinum poseidoni*) and the seep habitat contains higher levels of sulfide and methane than the habitats of non-seep specialist frenulates [[35], [36], [37], [38]], it was possible that *O. haakonmosbiensis* was specialized for the seep environment of HMMV. However, the species description of *O. haakonmosbiensis* was subsequently questioned because the fjord frenulate *Oligobrachia webbi* [39] was morphologically very similar. A revision was then published, upholding *O. haakonmosbiensis* as a separate species, and describing minor but distinct morphological differences from *O. webbi* [40]. Subsequent genetic work has demonstrated that at least three *Oligobrachia* species inhabit Arctic cold seeps. As mentioned above, *O. haakonmosbiensis* is one; this species is present at sites in the Norwegian Sea and the Fram Strait, as well as at subarctic sites such as the Nyegga-Storegga slide [[34], [40], [44]]). The *Oligobrachia* sp. CPL-clade (or more simply, CPL-clade) gets its colloquial name from the crater site in Bjørnøyrenna (Barents Sea), the pingo site in Storfjordrenna (Barents Sea) and seeps in the Laptev Sea where it was first recorded [41,42]. This species also inhabits mud volcanoes in the Beaufort Sea [43]. Finally, an undescribed species has been documented at the Vestnesa seep site in the Fram Strait, which we hereafter refer to as *Oligobrachia* Vestnesa [44]. For images and descriptions of the morphology of these cryptic species of *Oligobrachia*, the reader is referred to publications by Sen et al. [41,44] and Smirnov [34,40]. Whether *O. webbi* was identical to one of these three species remained an unresolved question since no DNA sequence data were available for *O. webbi*, due to the species being described before the development of genetic tools and molecular methods.

In addition to host species identity, the nutrition and specific mode of chemosynthesis among Arctic seep *Oligobrachia* has been debated. Frenulates as a group have an obligate symbiotic relationship with bacteria from whom they derive the bulk of their nutrition, but they are known to host both methane and sulfide oxidizing symbionts [[45], [46], [47]]. Based on carbon stable isotope ratios, the HMMV species of *Oligobrachia* was suggested to host methane-oxidizing symbionts [48,49]. However molecular and microscopic methods failed to detect these, and instead provided evidence for sulfide oxidizing symbionts [41,50]. Therefore, though *Oligobrachia* frenulates are present in both fjord and seep ecosystems in high latitude regions, fundamental questions regarding their phylogeography and ecological niches remain. Since these animals, through their chemosynthetic partners directly generate animal biomass on the seafloor, and alter sediment geochemistry, these questions are pertinent not only to ecosystem functioning, but are additionally linked to larger-scale patterns, such as distributions of chemosymbiotrophic fauna in the Arctic and whether carbon, energy and nutrient flows in the Arctic have chemosynthesis-based pathways that have largely been unaccounted for till date.

This study was aimed at targeting these unresolved questions regarding the phylogeography and nutritional ecology of high latitude *Oligobrachia.* We determined the genetic identity of *O. webbi*, which we achieved by revisiting and resampling the type locality of the species (Kvalsund fjord/sound off Tromsø, northern Norway), and by applying ancient DNA (aDNA) extraction methods to individuals from the original museum voucher material. We also performed stable carbon, nitrogen and sulfur isotope analyses and conducted sediment porewater geochemical profiles on Kvalsund samples in order to understand the ecology of these chemosymbiotrophic animals in a non-seep environment. We additionally included genetic work, sediment porewater chemistry analyses and stable isotope analyses for other Arctic frenulates and sites to expand the scope of our study. Through this multifaceted approach, we aimed to not simply reveal the genetic identity of a specific species, but to additionally tackle fundamental ecological concepts regarding chemosynthesis and Arctic marine ecology.

## 2 Materials and methods

### 2.1 Museum samples

The original type material of *O. webbi* at the time of its description by Brattegard [39] was stored as three collections at two museums in Norway, originally fixed in 2% formaldehyde and subsequently transferred to 70% ethanol. Collection numbers 47 977 and 47 978 were stored at the Museum of Zoology, University of Bergen, and consist of a female holotype and assorted body parts respectively. Collection number P 746, stored at the Museum of Tromsø (now the Arctic University Museum of Norway), consists of various body parts [39]. We obtained one individual (various parts of the tube and animal) from the Arctic University Museum collection in October 2020 courtesy Andreas Altenberger and Inger Greve Alsos.

### 2.2 New samples from the type locality

*O. webbi* was described from Kvalsund (Tromsø, Norway) in 1966, which predates modern GPS technology. However, Brattegard [39] provided approximate coordinates of the location and details, that we used to guide field collections in October 2020. The site was described as being northwest of Gåsvær (69° 57’N, 18° 34’E), off Kvaløya in the Kvalsund sound near Tromsø, at a water depth of 270 m, in sandy mud. Using the multibeam echosounder and the chirp system on board the R/V *Helmer Hanssen*, we narrowed down specific locations for taking core samples with the coordinates provided, in order to replicate the original sampling location as much as possible. At the type locality of *O. webbi,* first, CTD (Conductivity, Temperature and Depth) measurements were taken with a Seabird 911 Plus to obtain basic water column parameters. Two box core samples and one Van Veen grab were then taken to collect new worm specimens. Sediment was sieved over a 1-mm mesh and recovered individuals were either frozen immediately within their tubes or kept in the dark and cold until they could be extracted from their tubes (not more than a few hours). The latter group of specimens were carefully extracted from their tubes with a pair of fine forceps and paintbrush and then either frozen or preserved in absolute ethanol.

In addition to new topotype specimens, porewater and sediment samples were taken for analyzing porewater chemistry and sediment elemental content (see Table 1 for an overview of the samples taken for this study). Subcores within the two box cores, and a gravity core were taken to obtain these samples. Two subcores were taken from each box core: one was drilled with 2.5-cm sized holes every 2 cm for pore water sampling, and one with 2.5 cm holes every 5 cm for taking methane and total organic carbon (TOC) samples. Holes were taped before pushing the cores into the sediment. The gravity core was sampled for dissolved methane, TOC, and pore water, and was drilled with 2.5 cm wide holes every 10 cm and taped before coring.

**Table 1:** Overview of the samples used in this study. Note that this table lists only samples/data used for the first time in this study (e.g., additional samples have been taken at the LV canyon site that have been published earlier and are referenced in this study).

Site	Sampling gear	Year	Latitude (°N)	Longitude (°E)	Samples/purpose
Kvalsund	CTD	2018	69° 56.26'	018° 35.08'	water column temperature
Kvalsund	CTD	2020	69° 56.48'	018° 35.06'	water column temperature
Kvalsund	Box core (640BCE)	2020	69° 55.88’	018° 35.73’	porewater sulfide (subcore)
					porewater methane (subcore)
					TOC
					frenulates (DNA)
					frenulates (stable isotopes)
Kvalsund	Box core (643BCE)	2020	69° 05.16’	018° 35.32’	porewater sulfide (subcore)
					porewater methane (subcore)
					TOC
					frenulates (DNA)
					frenulates (stable isotopes)
Kvalsund	Gravity core (641 GC)	2020	69° 56.04’	018° 35.31’	porewater sulfide
					porewater methane
Kvalsund	Van Veen grab	2020	69° 55.92’	018° 35.48’	frenulates (DNA)
					frenulates (stable isotopes)
Nordfjord	Van Veen grab	2020	67° 07.46'	014° 016.42'	TOC
					frenulates (DNA)
					frenulates (stable isotopes)
LV canyon seep	ROV push core	2020	68° 10.02'	010° 28.20	frenulates (stable isotopes)

Porewater was retrieved using rhizons (every 2 cm for subcores from the box cores, and every 10 cm for the gravity core) connected to 10 mL syringes with wooden sticks for suction. Bottom water, i.e., water just above the sediment surface was collected in addition to porewater. The first 1–1.5 mL pore water was discarded. Thereafter 1 mL was put in Eppendorf vials pre-prepped with 1 mL Zinc acetate (19.6 mM) for porewater analysis. These were frozen at −20 °C.

Sediment plugs (3 mL in volume) were taken for measuring sediment methane concentration i.e., headspace (3 mL in volume) and TOC (20 mL in volume, every 5 cm from the two subcores from the boxcores and every 10 cm for the gravity core).

### 2.3 Methane and porewater measurements

The 3 mL sediment plugs for methane were transferred to glass vials containing 3 mL 5% NaOH solution. The vials were immediately crimp sealed with butyl rubber stoppers, shaken to suspend the sediment plug and stored cold at 4 °C. Methane concentrations were measured with a gas chromatograph (GC, SRI Instruments 310C) with a 0.9 m packed silica gel column and a flame ionization detector. Upon analysis samples were shaken to equilibrate the methane with the headspace of the vial. Then 500 μL of the headspace was collected with a glass syringe-needle and injected into the GC. Methane standards were prepared by injecting volumes (between 0.5 and 2 mL) of pure methane gas into 117 mL glass bottles sealed with butyl rubber stoppers. Laboratory air contained 3 ppm methane, which was considered the lower detection limit of the analyses.

The concentrations of total dissolved sulfide (ΣHS = H_2_S + HS^−^ + S^2−^) were determined by the iodometric method (US Environmental Protection Agency, method 9030 and [51]). Before the analyses, samples were centrifuged for 5–7 min at 2000 rpm to separate the ZnS precipitates from the residual porewater. The supernatant fluid was pipetted and discarded as it may contain other reductants (such as dissolved organic carbon species) that may react with I_2_ and affect the results. The remaining ZnS precipitates were washed into a glass beaker with ca. 2 mL of 18Ω Milli-Q water for titration. Iodine (I_2_) solution of ca. 14 mM was added (0.2 mL). Aliquots of starch solution (0.05 mL; prepared every other day) was added as an end-point indicator and 4 M HCl was added to ensure a completed reduction of I_2_ to 2I^−^ by lowering the sample pH with HCl [51]. The ZnS in the sample then reduces I_2_ when in contact (ZnS + I_2_ → 2ZnI + S). We then titrated the residual I_2_ to calculate the amounts of total sulfide in the samples (i.e. I_2-unreacted_ – I_2-residual_ = ZnS_sample_). Factory-made 0.00109 N Na-thiosulfate (stabilized standard solution, Hach Lot# 2408949) was sequentially diluted 10 times and 100 times and used as titrants. Titrants were added to the sample with an automatic pipette under constant mixing in an open beaker until the purple color faded away as a result of complete I_2_ reduction. The amounts of titrant were then recorded for the calculation of ZnS_sample_. As I_2_ is fairly unstable when exposed to light, its concentration was closely monitored every ca. 30 min during the titration to constrain I_2-unreacted_. The uncertainty of the measurements was then determined from two closest I_2_ measurements before and after the titration of the actual sample. In general, the concentration of I_2-unreacted_ decreased by 0.27 mM every hour. New I_2_ was used during the same session of analyses if the I_2-unreacted_ concentration was below 85% of its concentration earlier in the session. The Zn-acetate solution used to precipitate out total sulfide was also titrated following the identical protocol to ensure no measurable sulfide in it.

### 2.4 Stable isotope analyses and elemental content measurements

On board, animals and sediment samples for stable isotope and elemental content analysis were frozen at −20 °C. Once back on land, they were freeze-dried and stored in airtight containers. Whole individuals of the worms were analyzed due to their small body size (as opposed to cutting off a piece of the animals). Sediment aliquots were ground to a homogeneous powder using mortar and pestle and acidified to remove carbonates by direct addition of excess 1 M HCl in small increments, and subsequently rinsed with distilled water. Sediment samples were analyzed twice: once using acidified material (for C elemental content and stable isotope ratios) and once using native material (for N and S elemental contents and stable isotope ratios).

Elemental content was measured using a vario MICRO cube C–N–S elemental analyzer (Elementar Analysensysteme GMBH, Hanau, Germany) as relative percentage of analyzed mass (mass%). Since carbon content was measured on acidified sediments (i.e., after carbonate removal), it can be considered a proxy of total organic carbon (TOC) content. Empty tin cups were used as analytical blanks. Sulfanilic acid (Sigma-Aldrich; %C = 41.6%, %N = 8.1%, %S = 18.5%) was used as the elemental standard.

Stable isotope ratio measurements were performed via continuous flow – elemental analysis – isotope ratio mass spectrometry (CF-EA-IRMS) at University of Liège (Belgium), using the abovementioned vario MICRO cube C–N–S elemental analyzer coupled to an IsoPrime100 isotope ratio mass spectrometer (Isoprime, Cheadle, United Kingdom). Isotopic ratios were expressed using the conventional δ notation [52], in ‰ and relative to the international references Vienna Pee Dee Belemnite (VPDB) (for carbon), Atmospheric Air (AIR) (for nitrogen) and Vienna Canyon Diablo Troilite (VCDT) (for sulfur). IAEA (International Atomic Energy Agency, Vienna, Austria) certified reference materials sucrose (IAEA-C-6; δ^13^C = −10.8 ± 0.5‰; mean ± SD), ammonium sulfate (IAEA-N-2; δ^15^N = 20.3 ± 0.2‰; mean ± SD) and silver sulfide (IAEA-S-1; δ^34^S = −0.3‰) were used as primary analytical standards. Sulfanilic acid (Sigma-Aldrich; δ^13^C = −25.6 ± 0.4‰; δ^15^N = −0.13 ± 0.4‰; δ^34^S = 5.9 ± 0.5‰; means ± SD) was used as a secondary analytical standard. Standard deviations on multi-batch replicate measurements of secondary and internal lab standards (seabass muscle and coastal sediments from the Bay of Brest) analyzed interspersed with samples (one replicate of each standard every 15 analyses) were 0.1‰ for δ^13^C, 0.2‰ for δ^15^N, and 0.4‰ for δ^34^S.

### 2.5 DNA work and species identification

Storage in formaldehyde degrades nucleic acids and makes DNA extraction difficult. Therefore, we applied the QIAamp DNA FFPE Tissue Kit (Qiagen) to extract DNA from the museum sample following the manufacturer’s protocol with a few modifications. Specifically, the paraffin-removing step was not conducted. The lysis procedure was performed overnight and finally RNA-carrier was added to the AL buffer to enhance DNA recovery.

For the new topotypes from the *O. webbi* type locality (Kvalsund), whenever possible, worms were extracted from their tubes before proceeding to DNA extraction. If extraction from tubes was not possible, the whole animal and its tube were ground together during the tissue lysis from which DNA was extracted. DNA extraction was performed with the Dneasy Blood and Tissue Kit (Qiagen) following the manufacturer’s protocol.

For species identification the mitochondrial cytochrome oxidase subunit one (mtCOI) was amplified using the universal primers HCO2198 and LCO1490 [53]. We expected to obtain very low DNA concentrations as well as fragmented DNA from the museum sample. Consequently, we constructed three overlapping primer sets from the alignment of *O. haakonmosbiensis* and *Oligobrachia* Vestnesa which was used to amplify the complete, 668 bp-long COI gene. The first primer set (primCOI1_F: ATC TGA GTT GGA CTA ATT GC; primCOI1_R: ACT AAA AGA ATT ACT GCA GGA) amplified ∼269 bp, the second primer set (primCOI2_F: TCC TGC AGT AAT TCT TTT AGT; primCOI2_R: TTA ACG AAG TCC TTT ATA TCG) amplified ∼217bp and the third primer set (primCOI3_F:CGA TAT AAA GGA CTT CGT TTA; HC02198) amplified ∼218 bp.

DNA amplification for species identification of the samples with the prefix ‘M’ (Table 2) was conducted at the Department of Biosciences and Aquaculture, Nord University, Bodø, Norway. The PCR conditions for this amplification were: 1 × 95 °C: 10 min, 38 × 95 °C: 1 min, 52 °C:1 min, 72 °C: 1 min followed by 72 °C: 7 min. Successfully amplified products were consequently sequenced on a 3500xL Genetic Analyzer (Applied Biosystems).

**Table 2:** Species identification of worms sampled in this study. Lab/ID refers to the lab name for each sample, with W indicating that the sample was processed at Aarhus University, Denmark, and M referring to that sample having been processed at Nord University, Norway.

Site	Lab/ID	Species identification	GenBank results^a^
Kvalsund	W1	*Siboglinum fiordicum*	W1
Kvalsund	W2	*Siboglinum fiordicum*	W1
Kvalsund	W5	*Siboglinum fiordicum*	MK673144.1
Kvalsund	W7	*Siboglinum fiordicum*	MK673144.1
Kvalsund	W8	*Siboglinum fiordicum*	MK673144.1
Kvalsund	W9	*Siboglinum fiordicum*	MK673144.1
Kvalsund	W10	*Siboglinum fiordicum*	MK673144.1
Kvalsund	W11	*Siboglinum fiordicum*	W11
Kvalsund	W12	*Siboglinum fiordicum*	W11
Kvalsund	W13	*Siboglinum fiordicum*	W11
Kvalsund	W15	*Oligobrachia haakonmosbiensis*	W15^b^
Nordfjord	W16	*Siboglinum fiordicum*	MK673144.1
Nordfjord	W17	*Siboglinum fiordicum*	W17
Nordfjord	W18	*Siboglinum fiordicum*	W17
Nordfjord	W19	*Siboglinum fiordicum*	W17
Nordfjord	M12	*Siboglinum ekmani*	KF444429.1
Nordfjord	M15	*Siboglinum ekmani*	KF444429.1
Nordfjord	M20	*Siboglinum ekmani*	M20^c^
Nordfjord	M21	*Siboglinum ekmani*	M20
Nordfjord	M22	*Siboglinum ekmani*	M22
Nordfjord	M25	*Siboglinum ekmani*	KJ789169.1
Nordfjord	M26	*Siboglinum ekmani*	M26
Nordfjord	M29	*Siboglinum ekmani*	KF444429.1
Nordfjord	M30	*Siboglinum ekmani*	KF444429.1
Nordfjord	M31	*Siboglinum ekmani*	KF444429.1
Nordfjord	M33	*Siboglinum ekmani*	KF444429.1
Nordfjord	M34	*Siboglinum ekmani*	KF444429.1
Museum	W20	*Oligobrachia webbi*	O_webbi

a 100% match based on 574bp.

b 100% match with MH619663 (*O. haakonmosbiensis*, Sen et al., 2018) based on 410bp.

c 100% match with KJ789169.1 based on 410bp.

DNA amplification for species identification of samples with prefix ‘W’ (Table 2) was conducted at the Department of Biology, Section for Microbiology, Aarhus University, Denmark. The PCR conditions in this case were: 1 × 95 °C: 1 min, 35 × 95 °C: 45s, 52 °C: 45s, 72 °C: 20s followed by 72 °C: 10 min. The resulting PCR products were sequenced at MACROGEN Europe. The following species identification based on the sequence alignment was performed in Sequencher 5.3 (GeneCode) and the website National Center for Biotechnology Information (NCBI), and Basic Local Alignment Search Tool (BLAST) (http://blast.ncbi.nlm.nih.gov/Blast.cgi).

### 2.6 Phylogeny inferences

The following COI sequences (KJ789171, FJ480370, FJ480373, FJ480374, FJ480376, FJ480377, FJ480398, FJ480399, FM178481, FM178482, KJ789170.1, MH619658, MH619661, MH619662, MH619663, MH619665, MH619668, MH619669, MH619671, MH619674, MK673145.1, MK673146, MK673149, MH619659, MH619686, MK673143, MK673144.1, MK673150, MK67315) representing *Spirobrachia* sp., *Siboglinum fiordicum*, *Oligobrachia haakonmosbiensis*, *Oligobrachia* sp., *Oligobrachia* Vestnesa, *Spirobrachia tripeira*, *Lamellisabella denticulate*, *Spirobrachia* cf*. grandis* and *Siboglinum poseidonii* were downloaded from GenBank and aligned in Sequencer 5.3 (GeneCode) together with the observed haplotypes from the present study. Downloaded sequences were cropped to 574 bp and 410 bp, following sequences obtained in the present study, and resulting haplotypes were identified in DnaSp. A phylogenetic consensus tree was inferred for the identified haplotypes, using the Bayesian method implemented in MrBayes v3.2.6 [54] based on a GTR model (best fit from jModelTest; [55]) (data not shown). MrBayes was run using the default parameter values (sample frequency = 500, diagnostic frequency = 5,000, ngen (run length) = 1 000 000), running two independent MCMC runs and discarding 25% of the samples of burn-ins. The analysis was stopped when standard deviation of split frequencies was below 0.01, indicative threshold of convergence [54]. The obtained topology and branch lengths of the tree were visualized in FigTree (http://beast.bio.ed.ac.uk/software/FigTree).

### 2.7 Additional sampling

In addition to the CTD data collected from the Kvalsund type locality of *O. webbi* during our sampling campaign in October 2020 with the R/V *Helmer Hanssen*, CTD data from the same location was retrieved from a prior cruise with the R/V *Johan Ruud* in February 2018, to compare seasonal differences in bottom water temperature.

**Fig. 1:**
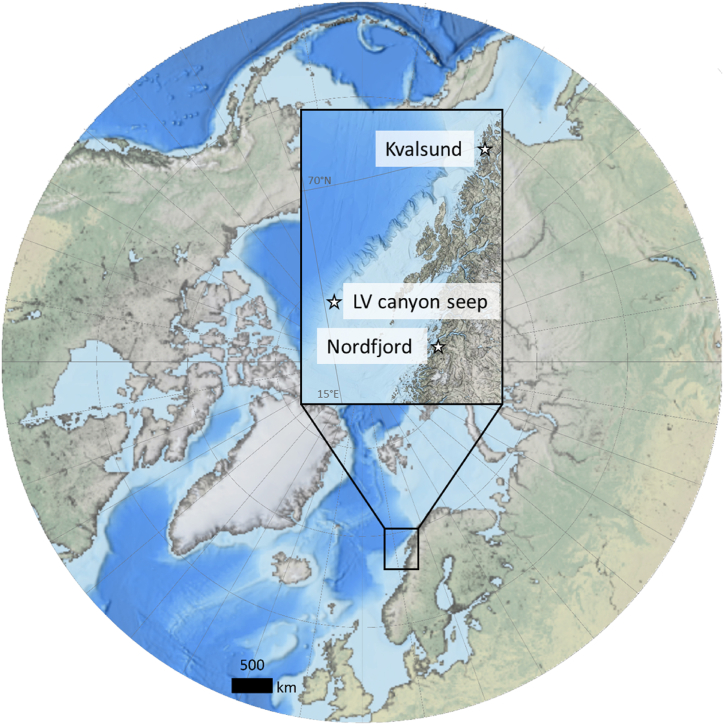
The circum-Arctic with the sites in this study highlighted.

In addition to the topotypes, samples of frenulate worms were collected from the Lofoten-Vesterålen (LV) canyon seep site [56,57] and Nordfjord off northern Norway (Fig. 1, Table 1). The LV canyon is a site of active methane seepage, with characteristic features such as methane-derived authigenic concretions and extensive mats of filamentous microbes [57]. Nordfjord and Kvalsund, on the other hand, represent fjord/sound or non-seep environments where methane and subsequent sulfide generation is likely due to the accumulation and decomposition of marine and terrestrially derived phytodetrital material. Both DNA work and stable isotope analyses were performed on individuals from the additional locations of the LV canyon seep and Nordfjord, following the same methodologies as described above. The LV canyon seep frenulates were earlier identified as *O. haakonmosbiensis* and carbon and nitrogen stable isotope values were measured for them as well [57], however, sulfur isotope analyses had not been conducted before this study. Neither DNA nor isotope work have been conducted on Nordfjord frenulates prior to this study. Sediment samples for carbon and nitrogen content were also taken from Nordfjord similarly to samples from Kvalsund, however, since a Van Veen grab was used for sampling at Nordfjord, only surface samples were taken (see Table 1 for an overview of all the sampling conducted as part of this study).

## 3 Results

### 3.1 Worm species identities

**Fig. 2:**
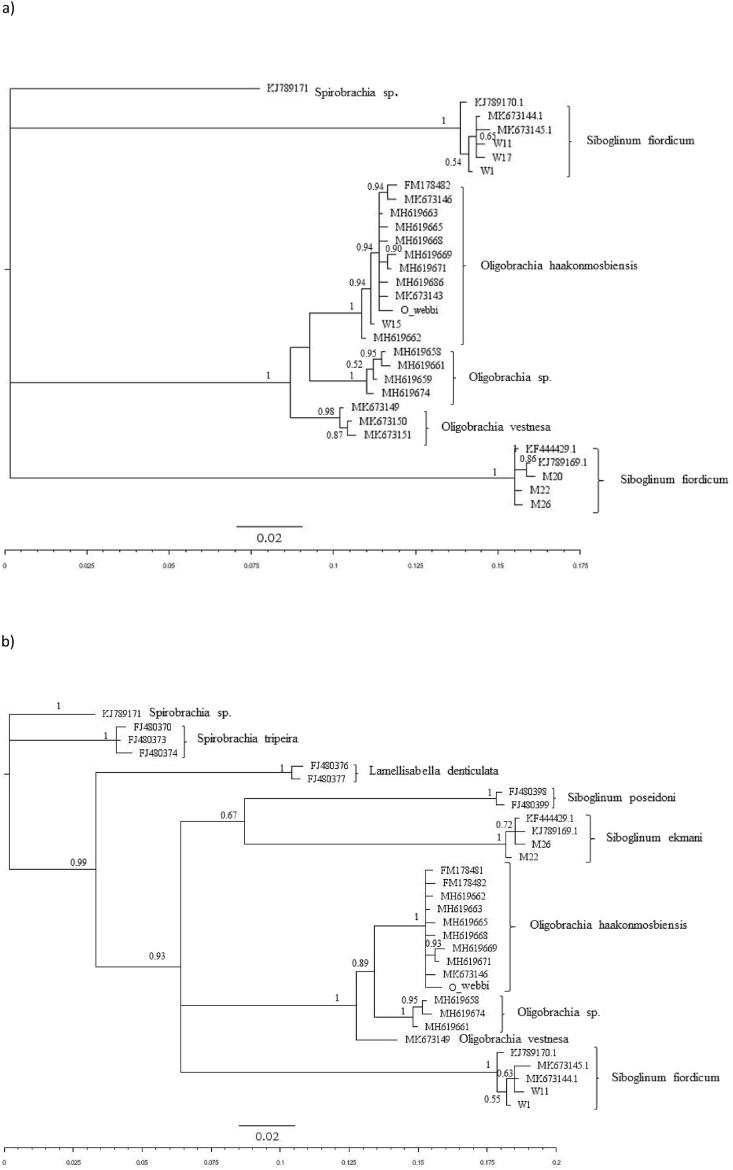
Bayesian phylogenetic tree (MrBayes) based on 574 bp (a) and 410 bp (b) of the COI sequences obtained from the different *Oligobrachia* frenulate species (see Sen et al. [41,44,57], Brattegard [39] and Smirnov [34,40] for images and descriptions of the different species of *Oligobrachia*). Values shown at each node represent the posterior probability.

The mitochondrial COI gene (574 bp) was successfully amplified and sequenced from the single *O. webbi* museum specimen and all the Kvalsund and Nordfjord specimens used in this study. All specimens sequenced in this study fall within the frenulate clade (Fig. 2 a,b). Among the Nordfjord specimens, we obtained a 100% match for two distinct species; *Siboglinum ekmani* and *Siboglinum fiordicum* (Fig. 2 a,b)*.* Though we did not conduct species level morphological identifications on collected samples, overall, the *Siboglinum* genus is characterized by having a single, non-pinnule bearing tentacle. All except one specimen from Kvalsund also exhibited this morphology and were a 100% match for *Siboglinum fiordicum* (Fig. 2 a, b)*.* The one specimen from Kvalsund that clearly differed from *Siboglinum* had multiple tentacles and instead resembled *Oligobrachia haakonmosbiensis.* Indeed, we obtained a 100% match with *O. haakonmosbiensis* for this individual. The museum sample also fell within the *O. haakonmosbiensis* clade. In short, the original type material of *O. webbi* was genetically the same as *O. haakonmosbiensis*, as was the one multi-tentacled individual recovered during our 2020 Kvalsund sampling campaign. All other samples from the two fjord locations belonged to a different genus (*Siboglinum*), with *S. fiordicum* and *S. ekmani* at Nordfjord and *S. fiordicum* at Kvalsund.

### 3.2 Stable isotope composition of the worms

We measured stable carbon, nitrogen, and sulfur isotope ratios of *O. haakonmosbiensis* specimens from the LV canyon seep site (7 individuals), *S. fiordicum* and *S. ekmani* from Nordfjord (11 individuals; note that we could not distinguish between the species when they were frozen for isotope analyses) and of *S. fiordicum* from Kvalsund (12 individuals).

**Fig. 3:**
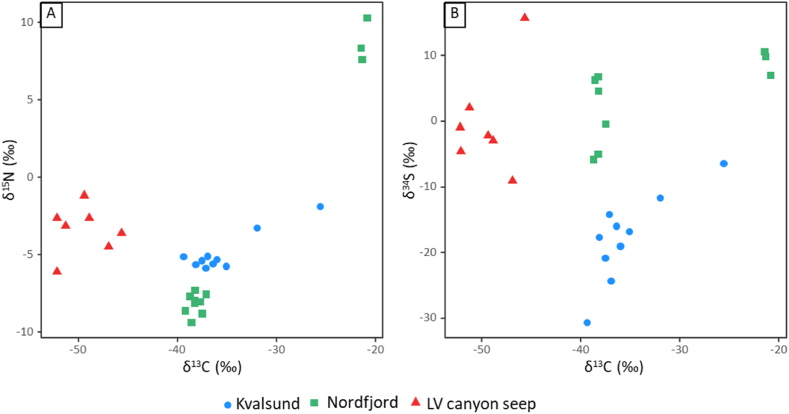
Biplots of ẟ^13^C and ẟ^15^N (A) and ẟ^13^C and ẟ^34^S (B) of worms from Kvalsund, Nordfjord and the LV (Lofoten-Vesterålen) canyon seep. Different species were present at the different sites: *Siboglinum fiordicum* at Kvalsund, *Siboglinum ekmani* and *Siboglinum fiordicum* at Nordfjord, and *Oligobrachia webbi* at the LV canyon seep.

The *O. haakonmosbiensis* LV seep samples had the lowest δ^1^³C values, ranging from −52.2‰ to −45.7‰ (Fig. 3 a, b). Values for individuals of the two *Siboglinum* species from the two fjord/sound sites were higher: among the *S. fiordicum* samples from Kvalsund, nine values out of 12 were lower than −35.0‰ which is normally associated with chemosynthesis [58], and one was also relatively low for photosynthesis (−32.0‰) [58] (Fig. 3 a,b). Two *S. fiordicum* samples from Kvalsund, however, had higher values in the range most often associated with photosynthesis (−26.9‰ and −25.6‰). The *S. fiordicum* and *S. ekmani* samples from Nordfjord displayed a similar trend, whereby most δ^1^³C values were lower than −37.0‰, but two individuals gave considerably higher values of −21.4‰ and −21.3‰. Overall, δ^1^³C values of the *Siboglinum* samples from the two fjord/sound sites were very similar, but higher than the values from LV canyon *O. haakonmosbiensis* seep site samples (Fig. 3 a,b).

Each group of samples (Kvalsund, LV and Nordfjord) displayed a fairly wide range of δ^1^⁵N values (Fig. 3a), and in theory, the values cross different trophic levels or indicate different degrees of assimilation of ammonium and nitrate [58]. Nordfjord samples (*S. fiordicum* and *S. ekmani*) exhibited the largest differences in δ^1^⁵N values across individuals, where most individuals (and all specimens with δ^1^³C values less than −37‰) had values ranging from about −9‰ to −7‰, while the two individuals with high δ^1^³C values had δ^1^⁵N values of 7‰ and 8‰. Most Kvalsund samples (*S. fiordicum*) had δ^1^⁵N values around −5‰, except the three individuals with higher δ^1^³C values, which had δ^1^⁵N of −3.7‰ and 1.8‰ for two of them. Nitrogen stable isotope ratios could not be measured in the last ^13^C enriched *S. fiordicum* worm from Kvalsund. In the LV seep *O. haakonmosbiensis* samples, δ^1^⁵N values ranged from −6‰ to −1‰ (Fig. 3a).

Kvalsund samples (*S. fiordicum*) overall had the lowest δ³⁴S values (Fig. 3b). The most ³⁴S-enriched signal was from an LV *O. haakonmosbiensis* seep sample, that had δ³⁴S in the range of methanotrophy or photosynthesis (15.8‰) [58].

### 3.3 Sediment porewater and elemental composition

**Fig. 4:**
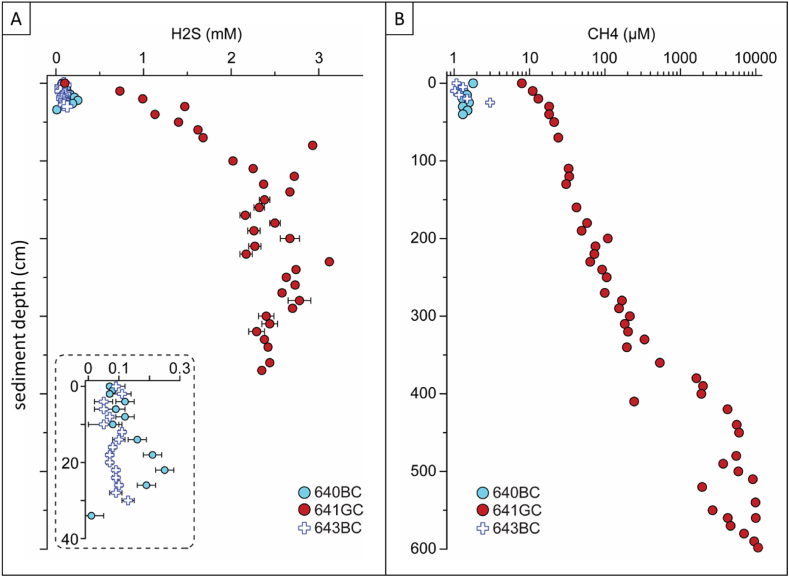
Sediment porewater profiles of sulfide (A) and methane (B) for the Kvalsund site. 640BCE = subcore from the first box core, 641 GC = gravity core, 643BCE = subcore from the second box core.

We measured sediment porewater concentrations of sulfide in the samples from Kvalsund (porewater sulfide has been measured at the LV site, see [[56], [57]]). Bottom water samples from Kvalsund contained sulfide in concentrations in tens of μM across the three cores (the two sub cores from the box cores, and the gravity core) and concentrations at the sediment-water interface were of similar range in the sub cores but reached 735 μM in the gravity core (Fig. 4a). Sulfide concentration did not vary much with depth in one of the sub cores from the box core, but both the other sub core and the gravity core displayed downcore increases in sulfide concentrations. In summary, high sulfide concentrations were measured both in the sediment as well as in the bottom water at Kvalsund.

Sediment porewater methane concentrations displayed somewhat similar trends to sulfide, i.e., tens of μM in shallower sediment depths, with a consistent increase, up till mM concentrations at around 5 m depth (Fig. 4b).

Total organic carbon (TOC) and total nitrogen (TN) were measured in the sediment samples from Kvalsund and Nordfjord (Table 3). Except for a few measurements, most TOC measurements at Kvalsund were above 10 mg/g, with levels reaching as high as above 100 mg/g (Table 3). TOC/TN (which can also be referred to as C/N) mass ratios were quite high as well, ranging from 2.4 to 121.4 and on average 17.4, indicating a substantial terrestrial input. Three of the four samples from Nordfjord had TOC levels between 21 and 30 mg/g, but one sample had a much higher level, at 73.5 mg/g. TOC/TN ratios were lower on average than at Kvalsund, but nonetheless were relatively high, ranging from 8.6 to 23.7, which is indicative again of considerable terrestrial input.

**Table 3:** Sediment characteristics of the two fjord sites (Kvalsund and Nordfjord).

Core/sample	Location	sediment depth (cm)	δ^1^³C (‰)	δ^1^⁵N (‰)	δ³⁴S (‰)	TOC (mg/g)	TN (mg/g)	TOC/TN (C/N)
box core 1	Kvalsund	0	−21.9	5.9	14.2	28.7	6.6	4.4
	Kvalsund	5	−21.8	6.5	10.1	97.4	6.5	15.0
	Kvalsund	10	−21.6	6.9	6.5	114.5	6.3	18.2
	Kvalsund	15	−21.5	6.7	11.1	110.2	6.2	17.8
	Kvalsund	20	−21.4	6.9	3.8	112.1	5.5	20.4
	Kvalsund	25	−21.0	7.0	3.2	106.7	5.2	20.5
	Kvalsund	30	−22.1	7.3	5.9	96.7	4.7	20.6
	Kvalsund	35	−21.0	7.1	2.9	84.1	4.7	17.9
	Kvalsund	40	−21.8	6.8	13.6	100.8	6.5	15.5
gravity core	Kvalsund	0	−22.3	6.7	0.3	7.3	3.0	2.4
	Kvalsund	10		4.0		95.6	2.8	34.1
	Kvalsund	20	−24.1	8.8	2.2	14.9	1.1	13.6
	Kvalsund	30	−21.3	5.6	2.6	44.1	2.3	19.2
	Kvalsund	50	−21.7	8.9	−0.9	36.4	1.4	26.0
	Kvalsund	60	−21.1	9.3	4.4	17.6	1.3	13.5
	Kvalsund	70	−21.8	7.5	−0.6	20.9	1.4	14.9
	Kvalsund	80	−22.4	8.4	6.8	23.0	1.1	20.9
	Kvalsund	100	−22.1	9.4	0.4	42.9	2.0	21.5
	Kvalsund	110	−21.5	9.1	2.0	14.0	1.7	8.2
	Kvalsund	120	−22.0	7.3	2.0	7.5	2.7	2.8
	Kvalsund	130	−21.9	7.0		38.8	2.2	17.6
	Kvalsund	140	−22.7	7.9	2.0	7.4	1.7	4.4
	Kvalsund	150	−21.9	6.8		10.0	1.9	5.3
	Kvalsund	160	−21.0	8.9	4.4	37.5	1.7	22.1
	Kvalsund	170	−22.1	7.6		6.8	1.7	4.0
	Kvalsund	180	−20.8	9.1	5.4	17.6	1.6	11.0
	Kvalsund	190	−21.1	8.5		21.2	1.6	13.3
	Kvalsund	200	−21.8	7.7		15.5	1.7	9.1
	Kvalsund	210	−21.3	9.2	2.5	21.6	1.7	12.7
	Kvalsund	220	−21.7	9.1	3.8	15.5	1.7	9.1
	Kvalsund	230		7.5		93.6	1.6	58.5
	Kvalsund	240	−21.9	7.6		41.5	1.7	24.4
	Kvalsund	250	−21.4	7.4		26.6	1.7	15.7
	Kvalsund	260	−21.5	8.8		15.5	1.4	11.1
	Kvalsund	270	−21.5	8.0		15.3	1.6	9.6
	Kvalsund	280	−21.4	8.0		22.1	1.7	13.0
	Kvalsund	290	−21.4	9.1	2.3	17.1	1.5	11.4
	Kvalsund	300	−21.3	9.4	0.5	22.4	1.7	13.2
	Kvalsund	310	−21.4	9.1	−2.5	23.2	0.9	25.8
	Kvalsund	320	−21.4	8.1		22.5	1.7	13.2
	Kvalsund	330	−21.6	7.9		23.0	1.7	13.5
	Kvalsund	340	−21.8	7.8		24.4	1.8	13.6
	Kvalsund	350	−21.6	7.8	4.4	52.6	1.0	52.6
	Kvalsund	360	−22.0	8.2		7.8	1.9	4.1
	Kvalsund	370	−21.9	8.0		20.1	1.7	11.8
	Kvalsund	380	−21.6	8.7		16.8	1.4	12.0
	Kvalsund	390	−22.1	8.2	1.1	14.9	0.9	16.6
	Kvalsund	400	−22.6	10.2		4.6	0.9	5.1
	Kvalsund	400	−21.7	5.9	5.9	15.3	1.8	8.5
	Kvalsund	410	−22.1	7.8		7.7	1.3	5.9
	Kvalsund	420	−22.5	8.1	6.3	11.2	0.6	18.7
	Kvalsund	430	−21.2	9.2		86.6	1.0	86.6
	Kvalsund	440	−22.3	8.3	10.4	10.6	0.9	11.8
	Kvalsund	450	−21.8	9.4		11.0	0.9	12.2
	Kvalsund	460	−21.8	9.8		19.6	0.8	24.5
	Kvalsund	470	−21.6	7.9	0.4	6.5	1.4	4.6
	Kvalsund	480	−21.8	9.9		6.0	0.7	8.6
	Kvalsund	490		8.0	9.3	97.1	0.8	121.4
	Kvalsund	500	−22.0	10.7		7.3	0.5	14.6
	Kvalsund	510	−21.5	9.6		20.1	0.8	25.1
	Kvalsund	520	−21.7	9.8		7.3	0.7	10.4
	Kvalsund	530	−22.1	10.3		6.6	0.8	8.3
	Kvalsund	540	−21.6	9.9		6.6	0.8	8.3
	Kvalsund	550	−21.6	8.7	1.8	7.2	0.8	9.0
	Kvalsund	560	−22.4	10.0		8.2	0.8	10.3
	Kvalsund	570	−21.7	8.4	0.7	6.7	1.4	4.8
	Kvalsund	580	−22.3	9.6		6.6	0.9	7.3
	Kvalsund	590	−22.0	10.0		11.7	0.9	13.0
	Kvalsund	598	−21.0	9.3		26.1	0.9	29.0
box core 2	Kvalsund	0	−21.8	6.1	12.2	106.3	5.2	20.4
	Kvalsund	5	−21.7	6.3	10.3	99.5	5.1	19.5
	Kvalsund	10	−21.4	7.0	6.0	100.2	4.6	21.8
	Kvalsund	15	−21.5	7.2	1.4	33.4	3.9	8.6
	Kvalsund	20	−20.9	5.9	0.5	87.4	3.3	26.5
	Kvalsund	25	−21.1	7.0	−1.1	67.7	3.3	20.5
Van Veen grab	Nordfjord	surface	−21.2	6.7	7.2	21.7	2.5	8.7
Van Veen grab	Nordfjord	surface	−21.6	6.3	11.2	21.4	2.5	8.6
Van Veen grab	Nordfjord	surface	−21.3	6.7	12.1	27.8	2.5	11.1
Van Veen grab	Nordfjord	surface	−21.3	7.8	11.5	73.5	3.1	23.7

## 4 Discussion

### 4.1 *Arctic* Oligobrachia *species*

The discovery of chemosynthesis-based ecosystems (CBEs) such as hydrothermal vents, hydrocarbon seeps, organic food falls and other reducing habitats revealed the existence of unique fauna specialized to exploit the toxic riches of these systems [[2], [3], [4],^59^]. Commonly, taxa highly adapted for life at CBEs are absent from other ecosystems, even though different kinds of CBEs tend to share taxa. Thus, the first time an Arctic seep was examined (HMMV), it was assumed that the discovered *Oligobrachia* species was a new species that had not been encountered before. Nonetheless, the acceptance of this new species, *O. haakonmosbiensis* came into question due to its morphology being extremely similar to that of the fjord/sound siboglinid, *Oligobrachia webbi.* It even led to the original description being modified and specifically compared to the description of *O. webbi* in order to validate *O. haakonmosbiensis* as a unique species [40]. DNA could not contribute towards resolving the debate because sequencing had only been conducted on *O. haakonmosbiensis*; the discovery and collection of *O. webbi* were made before DNA technology had been developed.

With the expansion of Arctic seep research, new seep localities were studied and found to host extensive fields of *Oligobrachia* species*.* Confoundingly, these species of *Oligobrachia* matched the descriptions of both *O. webbi* and *O. haakonmosbiensis* [41]*.* This is because there are no clear, distinct features between the two species, instead the description separates them based on marginal differences in the sizes of identical features [40]. The collected species of *Oligobrachia* from these new Arctic seeps (e.g., Bjørnøyrenna craters, Storfjordrenna pingos, shallow seeps in the Laptev Sea, mud volcanoes in the Beaufort Sea) spanned the range of sizes for both *O. webbi* and *O. haakonmosbiensis*, therefore, based on morphology alone, they could equally represent either species [41]*.* DNA was invoked to at least compare with *O. haakonmosbiensis*, and mtCOI sequences formed a clade separate from *O. haakonmosbiensis,* suggesting a distinct species (0.88/100 branch support), and subsequently, this new *Oligobrachia* was referred to as *Oligobrachia* sp. CPL-clade (also referred to as simply the CPL-clade for short, with CPL representing an abbreviation of the common names of the sites from which they were originally recovered) [41]. Due to the absence of sequences of *O. webbi,* it could not be determined whether *O. webbi* and the CPL-clade were the same species, and additionally, since it became clear that the morphological differences between *O. webbi* and *O. haakonmosbiensis* were insufficient to distinguish them as separate species, the additional possibility arose that *O. webbi* could be the same as *O. haakonmosbiensis.*

Through the use of ancient DNA extraction methods and sequencing COI amplicons, we have shown that the original museum specimen of *O. webbi* collected from Kvalsund [39] is genetically the same as specimens till date, referred to as *O. haakonmosbiensis*; their 574 bp long amplified COI fragments share 99% pairwise identity (two mutations) with *O. haakonmosbiensis* voucher V VI-143-1-HMMV from HMMV [41]*.* Sampling from the type locality of *O. webbi* yielded only a single specimen belonging to a species of *Oligobrachia*, and that too was genetically the same as what has been referred to as *O. haakonmosbiensis* (99% COI gene sequence identity, one mutation, across 574 bp nucleotides, also with voucher V VI-143-1-HMMV from HMMV [41]*.* The combination of our results shows that both the museum sample and the new topotype are *O. haakonmosbiensis.* Thus, *O. haakonmosbiensis* and *O. webbi* can be synonymized based on very little (if any) morphological and genetic difference between the two. It can be argued that the use of a single gene, in this case, mtCOI might not be sufficient to discriminate between *O. haakonmosbiensis* and the original topotype of *O. webbi* sample. However, mtCOI is often used to discriminate among siboglinids, and it offers higher phylogenetic resolution than the 18S rRNA gene [43,45,60]. Furthermore, mtCOI is sufficient to differentiate between *O. haakonmosbiensis* and *Oligobrachia* sp. CPL-clade [41,43]. In fact, at this point, mtCOI sequences exist for specimens belonging to *Oligobrachia* from HMMV, the Nyegga-Storegga slide, the LV canyon seeps, the Vestnesa seep site in the Fram Strait, seeps in the Barents Sea, Beaufort Sea, and Laptev Sea, in addition to the *O. webbi* original topotype and the specimen belonging to a species of *Oligobrachia* we collected in 2020 from the *O. webbi* type locality. Across these different sites and studies, three clades emerge, *O. haakonmosbiensis,* the CPL-clade and *Oligobrachia* Vestnesa [41,43,44]. With this level of discrimination possible with mtCOI alone, it is unlikely that there is a hidden separation between only *O. webbi* and *O. haakonmosbiensis* that we are failing to uncover. Therefore, the match between the *O. webbi* topotype as well as the new topotype to *O. haakonmosbiensis* strongly suggest that *O. webbi* and *O. haakonmosbiensis* are synonymous. The name *O. haakonmosbiensis* therefore needs to be relegated to a synonym for *O. webbi* and henceforth, the original name of *O. webbi* is the most correct nomenclature for this species.

Arctic seeps therefore currently host three species of *Oligobrachia*: *O. webbi,* the CPL-clade and *Oligobrachia* Vestnesa. All three are morphologically essentially identical, and if they constitute separate species as proposed, then they represent a cryptic species complex [41,44]. The latter two species, i.e., the CPL clade and *Oligobrachia* Vestnesa are new species that have not yet been named. The different species generally do not co-occur, the one exception being the Vestnesa seep site, which hosts both *Oligobrachia* Vestnesa and *O. webbi.* What factors prevent these similar animals with similar symbioses from co-occurring is unclear, particularly since our results demonstrate that the species overlap in their water depth distributions, thereby precluding water depth as being a separating factor as earlier suggested [44]. More work is needed to identify why Arctic seeps are so well parsed out between distinct species of *Oligobrachia.* At non-seep locations, we found overlap between species at Nordfjord but not at Kvalsund where we only identified *S. fiordicum.* It is possible that there are successional dynamics at play; it has, for example, been suggested that *S. fiordicum* is a later successional species [35] and this could at least explain why at Kvalsund, over the past fifty years, there has been a transition from *O. webbi* to *S. fiordicum.* However, we, at Nordfjord, and others (e.g., [61]) have observed co-occurring populations of *S. ekmani* and *S. fiordicum* in fjords in mid and southern Norway. Therefore, it is difficult, with the current state of knowledge, to understand species interactions and dynamics of Arctic frenulates.

### 4.2 *Generalist chemosymbiotic species in theArctic*

Methane and sulfide are two common energy sources for chemosynthesis and since the carbon source differs between the two oxidation pathways (methane vs dissolved inorganic carbon respectively), determining which of the two carbon sources is used is key to understanding the biology of *Oligobrachia* species and by extension, the type of carbon cycling in the ecosystems they inhabit. Enzymatic processes involved in methanotrophy, or methane oxidation, and thiotrophy, or sulfide oxidation, lead to different net carbon isotopic fractionation, and therefore carbon isotope composition is often used to differentiate between the two processes. Specifically, δ^1^³C < -35‰ or −30‰ are often associated with Calvin-Bensen cycle based thiotrophy, and lower values tend to be associated with methanotrophy, e.g., less than −40‰, sometimes −45‰ [58,59,62]. However, when methane in a particular system itself has low δ^1^³C values, it can lower the values associated with thiotrophy because the oxidation of this methane generates a depleted dissolved inorganic carbon (DIC) pool. This scenario has been seen in both *O. webbi* and the CPL-clade: methanotrophic symbionts were initially proposed for them based on carbon isotope values, but molecular and microscopic data clearly point towards thiotrophic symbionts [[41], [43], [50]]. One transmission electron micrograph of potential methanotrophic symbionts has been published for the CPL-clade [63], but the image is highly zoomed in, does not mention if it was taken from the symbiont containing organ (the trophosome), and overall, is not convincing that the structures shown represent methanotrophic bacteria. One of our LV samples had a δ^34^S value of 15.7‰, which is highly divergent from the other values we obtained for the LV samples (−1‰ and lower). This could be due to nutritional input within this individual from either photosynthesis or methanotrophy. If one considers the δ^1^³Cvalue of this sample (−45.7‰) to represent chemosynthesis, then this sulfur isotope value could be suggestive of methanotrophy in this individual. However, it had the highest δ^1^³C among all the LV samples, which casts some doubt on this. To conclude, only robust evidence of thiotrophic symbionts in *O. webbi* and the CPL-clade has been presented.

This means that *O. webbi* depends on sediment sulfide for its nutrition. At seeps this is easily provided through the coupling of sulfate reduction to the anaerobic oxidation of methane escaping from subsurface reservoirs [64,65]. Fjords and sounds such as Kvalsund where *O. webbi* also exists, do not necessarily have such subsurface pools of gas. Is its presence in fjords/sounds indicative of these locations having similar conditions as seep sites? The bottom water in fjord basins has been documented as being oxygen-deprived [19], and there is even documentation of bacterial mats coating the surface of fjord sediment. We collected porewater from Kvalsund to explicitly investigate how similar or different the fjord/sound or non-seep habitat of *O. webbi* is compared to its seep environments. We measured surprisingly high concentrations of sulfide at Kvalsund (high micromolar and even millimolar), rivalling concentrations at seeps (Fig. 4 a,b) and distinct from other non-seep habitats commonly inhabited by frenulates [35]. Sulfide can be generated through sulfate reduction coupled to decomposition of organic matter, and fjords/sounds tend to accumulate organic material because they receive both marine and terrestrial inputs that get concentrated due to limited water exchange with the open ocean [16,17,20]. We measured high TOC levels at Kvalsund (Table 3), therefore it is likely that this is the mechanism at play at least there, resulting in the generation of considerable amounts of sulfide in the sediment despite the absence of a subsurface methane reservoir.

Therefore, in the Arctic, where fjords have been etched into coastlines from the waxing and waning of ice sheets, environments rich in methane and sulfide might not be restricted to systems such as seeps. Our results, of *O. haakonmosbiensis* being identical to *O. webbi* signifies that it is not a seep-specialist species. Thus, even chemosymbiotic species of Arctic seeps are not necessarily seep-specific. Note that fossils of potentially specialist fauna have been recovered from Arctic seep sites, some of which have been dated to be from as recently as the late Pleistocene or Holocene [[66], [67], [68], [69], [70], [71]]. There is a possibility that some of these might even be extant species that have simply not been collected yet due to research on Arctic seeps being relatively scarce. Nonetheless, to date, living specialist fauna have not been recovered from present-day Arctic seep ecosystems. This is an intriguing phenomenon that opposes the worldwide trend of seeps hosting specialist fauna. Indeed, one of the classic paradigms of vent and seep ecology is that they host specialist fauna, and subsequently, such systems tend to have fauna that are more closely related to each other than to other background benthic fauna. Note that vents and seeps do not exclusively host specialist fauna, however, with the exception of shallow water locations, at least some specialists tend to be present at seeps, particularly chemosymbiotic species [13,14]. Other than the few shallow water ones [42,72,73], and thereby for the majority of Arctic seep sites studied, the lack of specialist fauna represents a deviation from trends at seeps in other parts of the world. Even on geological timescales, a paucity of seep specialist fauna has been observed at Arctic seeps [71,[74], [75], [76], [77]]. Based on our sediment geochemistry data, we posit that the presence of environments such as fjords and sounds with sulfidic conditions which are particularly widespread at the Nordic Seas and Barents Sea margin could have led to more generalized chemosymbiotic species than specialist ones. Apart from *O. webbi,* frenulates overall are quite common in fjords; we recovered *S. fiordicum* and *S. ekmani* from Kvalsund and Nordfjord, and these species have been recorded even in very dense aggregations across different fjords [21,23,39,61]. Frenulates are not the only group of chemosymbiotic animals either; thyasirid bivalves are common Arctic benthic macrofauna that can also form symbiotic associations with chemosynthetic bacteria [22,78,79].

Thyasirids are not obligately chemosymbiotic, and different individuals within single populations can have different degrees of reliance on chemosynthetic partners versus filter feeding [[78], [79], [80], [81], [82]]. In other words, thyasirids can rely both on the direct consumption of organic matter and chemosynthesis for their nutrition. Among frenulates the relationship with chemosynthetic bacteria is obligate, but nonetheless, experiments have demonstrated that they are capable of absorbing sediment organic particles across their epidermis [27,28]. Uptake of organic matter across their bodies was in fact proposed for frenulates before the discovery of chemosynthesis when scientists were baffled by the lack of feeding and digestive organs in these animals [27,28]. Therefore, similar to thyasirids, it is possible that Arctic frenulates are to a certain extent, mixotrophic, and capable of supplementing chemosynthetic carbon fixation with the uptake of sediment organic substances. Heterotrophic bacteria have been sequenced from seep frenulates from the Gulf of Cadiz, which led to the suggestion that frenulates host heterotrophic bacteria in addition to chemosynthetic ones [83], however, direct evidence of such a dual symbiosis does not currently exist.

Our isotope results suggest that at least *Siboglinum* species from our two fjord/sound sites obtain some of their nutrition from organic matter that was fixed photosynthetically. Two Kvalsund samples (*S. fiordicum*) and two Nordfjord samples (*S. fiordicum* and/or *S. ekmani*) had δ^1^³C values that are well within the photosynthetic spectrum (>−28‰). Furthermore, those individuals had higher δ^1^⁵N ratios as well, indicating that they are higher up the food chain, as opposed to being at the base of the food chain if they were functioning as primary consumers depending primarily on chemosynthesis. One individual from Kvalsund (*S. fiordicum*) had a value of −32.0‰ and though this is slightly higher than what is normally considered associated with chemoautotrophy, is nonetheless quite low to represent photosynthesis alone [58,59]. The δ^1^⁵N value for this individual (−3.3‰) was also higher than specimens with δ^1^³C values < −35‰ (−5.1–5.9‰), which further point towards nutrition being a mixture of photosynthetic and chemosynthetic sources. Similarly, the *O. webbi* LV individual with the very distinct δ^34^S value could also be representative of photosynthetic inputs into its diet; that would, for example, explain the fact that this individual had the highest δ^1^³C value among the LV samples. Since individuals that were used for isotope analyses were not the same as those on which genetic work was conducted, it is not possible to determine whether the isotopic signatures of the worms are due to heterotrophic symbionts or whether the animals are using and somehow digesting organic matter in another manner. Nonetheless, both our nitrogen and carbon isotope data point towards some degree of a mixotrophic lifestyle among Arctic frenulates, even if we cannot determine the mechanistic pathways of that trait.

**Fig. 5:**
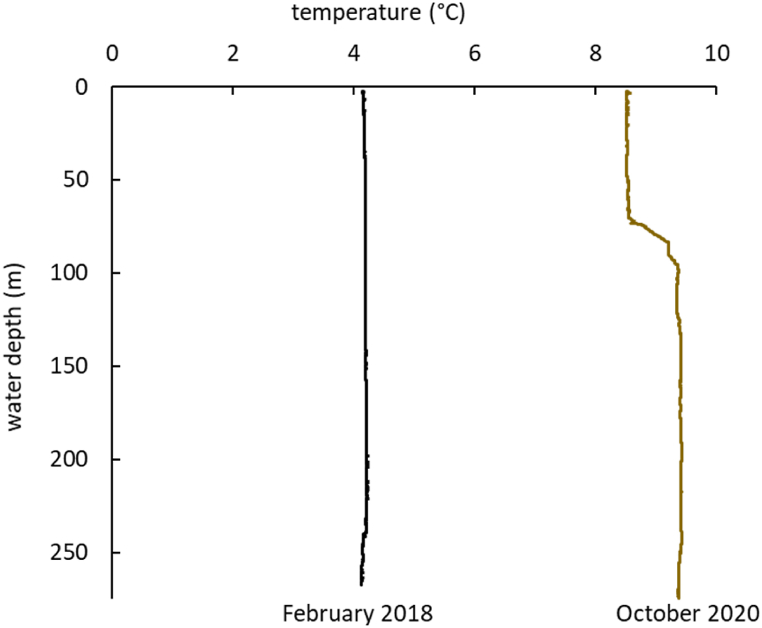
Temperature profile of the water column at the Kvalsund site, from CTD measurements, stations JR18-69CTD, February 1^st^, 2018, and HH20-644CTD, October 28^th^, 2020.

Therefore, chemosymbiotic fauna in the Arctic might be lacking specialization not only from a habitat point of view, but additionally might display some trophic plasticity as well. Arctic conditions may have influenced the nature of a non-specialized chemosymbiotic fauna in both seep and non-seep environments. Light regimes and the timing of surface blooms likely affect organic matter deposition in non-seep environments, and the subsequent rate at which sulfide is generated. Temperature strongly affects microbial activity, therefore, due to lower primary production and lower temperatures, sulfide generation rates in non-seep locations might decrease in the winter months. Indeed, our CTD data from Kvalsund demonstrate a drop in bottom water temperature from about 9 °C to 4 °C from October to February (Fig. 5), and February is not even the coldest month in northern Norway. Similarly, bottom water temperature on Arctic shelves, where methane seeps are increasingly being recognized as being common, vary considerably between seasons, with methane release at shelf seeps decreasing dramatically (by 43%) in colder, winter months [84]. Though temperature does not always impact gas hydrate dissociation [85], Mg/Ca of benthic foraminifera indicate that the gas hydrate stability zone can shift by at least 50 m water depth [86]. The latter case would result in extensive areas among seep sites having only a summertime exposure to sediment methane release. Aerobic methane oxidation rates also decrease at seeps, by an order of magnitude, from summer to winter months [87], and this trend, plus a decrease in methane release could mean that AOM rates plummet in the winter as well.

Thus, seasonal variations in sulfide fluxes likely exist in both seep and non-seep environments in the Arctic. Specifically, rates decline during the cold and dark winter months. Subsequently, in the Arctic winter, supplementing a chemosynthesis-based diet with photosynthetic organic matter uptake might be a useful strategy to counteract low sulfide generation rates. Similar to sulfide fluxes, sediment organic content would also decline in the dark winter months, but in contrast to what was thought before, the latter does not become non-existent during the polar night [88,89]. Relying more heavily on chemosynthesis during spring and summer bloom periods could actually be beneficial, since the rest of the benthos at that time would be competing intensively for relatively fresh phytodetrital organic matter. This strategy, of alternating the degree of reliance on sediment organic matter versus internal chemosynthetic production could be useful in the Arctic where strong seasonal dynamics are at play. This has been seen among thyasirids: symbiont abundances have been shown to decrease in thyasirids during the winter months, indicating seasonally induced differential degrees of reliance on symbiont-based chemosynthesis [82].

This strategy of seasonally shifting the degree to which symbionts versus the surrounding sediment provide nutrition might be important, and species that are adapted for seasonally variable OM input and temperature through mixotrophy instead of chemosynthesis alone might have an advantage in Arctic ecosystems. From a larger Arctic perspective, this implies that chemosynthesis is a part of Arctic marine food webs, not just at seeps or vents, but in other locations as well. In these other locations, photosynthesis and chemosynthesis are coupled to each other, specifically, sediment organic matter is derived from both chemosynthetic and photosynthetic production, and it is the degradation of this dually sourced sediment organic matter that generates the methane and sulfide that powers chemosynthesis. Terrestrial organic matter from catchments draining into fjords could contribute towards the photosynthetic component of fjord chemosynthetic production as well. In locations with sea-ice cover, as is common in Arctic fjords, sea-ice algal deposits to the benthos could represent yet another contribution to the organic matter fueling chemosynthesis-based processes. Sympagic-pelagic-benthic coupling is the Arctic archetype, with food or organic carbon flowing from the former to the latter, and nutrients and inorganic carbon flowing in the opposite direction. However, pathways might be more complex, with organic matter reaching the seafloor, being converted to inorganic carbon through remineralization as usual, but additionally being degraded to reduced compounds and then fixed again directly to animal biomass on the seafloor, with the subsequent transfer of both nutrients/inorganic carbon and organic carbon upwards from the benthos.

## 5 Conclusion

The glacial history of the Arctic has created fjords and sounds where highly reducing environments can develop, rivalling the conditions of methane seeps. We suggest that the presence of such sulfide and methane –rich systems outside of seeps has selected for chemosymbiotic species in various locations within the Arctic, which is potentially one explanation as to why Arctic seeps, unlike many other seeps in the world, lack a seep-specialist fauna. The presence of chemosymbiotic animals at both seeps and other benthic ecosystems in the Arctic suggests that nutrient and carbon transfer across realms in the Arctic might have steps that have been overlooked till now because the ubiquity of animals such as thyasirids and frenulates in the Arctic has not been explicitly examined within the context of chemosynthesis-based biology. At both seep and non-seep locations, chemosynthesis-based lifestyles could be strongly influenced by the sharp seasonality of the Arctic, and this likely results in chemosynthetic-photosynthetic coupling and seasonal dynamics to energy and carbon transfer that has not been considered within the Arctic marine ecosystem before.

## Author contribution statement

Arunima Sen; Tine L. Rasmussen: Conceived and designed the experiments; Performed the experiments; Analyzed and interpreted the data; Contributed reagents, materials, analysis tools or data; Wrote the paper.

Liselotte W. Andersen; Kasper U. Kjeldsen; Loïc N. Michel; Wei Li Hong; Marvin Choquet: Performed the experiments; Analyzed and interpreted the data; Contributed reagents, materials, analysis tools or data; Wrote the paper.

## Funding statement

This work was supported by 10.13039/501100005416Norges Forskningsråd [223259].

Wei Li Hong was supported by 10.13039/501100004359Vetenskapsrådet [2021-04962].

## Data availability statement

Data associated with this study has been deposited at GenBank SUB12100614 W1 (OP541390), SUB12100614 W11 (OP541391) SUB12100614 W15 (OP541392) SUB12100614 W17 (OP541393), SUB12100614 Webbi (OP541394), SUB12100614 M20 (OP541395), SUB12100614 M22 (OP541396), SUB12100614 M26 (OP541397).

## Declaration of interest’s statement

The authors declare no conflict of interest.
